# The complete plastid genome of *Iris domestica*: a traditional Chinese medicine

**DOI:** 10.1080/23802359.2019.1693923

**Published:** 2019-11-22

**Authors:** Hong-Lian Ai, Ke Ye, Xian Zhang, Xiao Lv, Zheng-Hui Li, Shu-Dong Zhang

**Affiliations:** a School of Pharmaceutical Sciences, South-Central University for Nationalities, Wuhan, China;; b School of Biological Sciences and Technology, Liupanshui Normal University, Liupanshui, China

**Keywords:** Plastid genome, *Iris domestica*, phylogenetic analysis

## Abstract

*Iris domestica* has been used as Chinese traditional medicine to treat inflammation and throat disorders for many centuries. In this study, the complete plastid genome of *I. domestica* was first reported and characterized. The complete plastid genome is a typical quadripartite circular molecule of 153,729 bp in length, including a large single copy (LSC) region of 83,136 bp and a small single copy (SSC) region of 18,165 bp separated by two inverted repeat (IR) regions of 26,214 bp. A total of 132 genes including 86 protein-coding genes, 38 tRNA genes, and eight rRNA genes were identified. The phylogenetic analysis revealed that *I. domestica* was closer to *I. gatesii*.

*Iris domestica* (L.) Goldblatt & Mabb. (Iridaceae), has long been used in traditional Chinese medicine and other East Asian phytotherapy systems as antipyretic agents, antidote, expectorant, antiphlogistic and analgesic (Woźniak and Matkowski 2015; Zhang et al. 2016). Recently, it has been reported that *I. domestica* exhibited high hypoglycemic activities and could be explored as possible therapeutic agents for type 2 diabetes mellitus (Guo et al. 2019). However, most of the studies for this species mainly focused on phytochemistry and pharmacological activity (Zhang et al. 2016; Song et al. 2018; Li et al. 2019), with little research on its molecular biology. In this paper, the complete plastid genome of *I. domestica* was characterized with the Illumina next-generation sequencing techniques.

Fresh leaves of *I. domestica* were collected from Longping town of Jianshi (Hubei, China; 30°48′24″N, 110°1′47″E, 1,750 m). The voucher specimen (HSN11621) was deposited in the herbarium of South-Central University for Nationalities (HSN). The total DNA of *I. domestica* was isolated using the modified CTAB method (Doyle 1987) and used for sequencing on the Illumina HiSeq 4000 Platform at the Beijing Novogene Bioinformatics Technology Co., Ltd (Nanjing, China). The plastid genome was *de novo* assemble using SPAdes (v 3.12.0) (Bankevich et al. 2012). The annotation of the complete genome was conducted using Dual Organellar Genome Annotator (DOGMA) (Wyman et al. 2004) with manual adjustments. The complete plastid genome of *I. domestica* (GenBank Accession Number: MN630999) represents a typical quadripartite circular molecule with 153,729 bp in length. It is composed by a large single copy (LSC) region of 83,136 bp, a small single copy (SSC) region of 18,165 bp and a pair of inverted repeat (IR) regions of 26,214 bp. The genome encodes 132 genes, including 86 protein-coding genes, 38 tRNA genes, eight rRNA genes. Fifteen genes (*atpF*, *ndhA*, *ndhB*, *petB*, *petD*, *rpl16*, *rpl2*, *rpoC1*, *rps16*, *trnA-UGC*, *trnG-GCC*, *trnI-GAU*, *trnK-UUU*, *trnL-UAA*, *trnV-UAC*) contain one intron and three genes (*clpP, rps12, ycf3*) contain two introns. The overall GC content of *I. domestica* plastid genome is 37.9%.

Because of its distinctive floral, fruit, and seed morphology, *I. domestica* had been placed in a separate genus *Belamcanda* as its sole species, *B. chinensis*. However, recent molecular DNA sequence evidence showed *B. chinensis* nested within the genus *Iris* (Goldblatt and Mabberley 2005). To confirm the phylogenetic position of *I. domestica*, phylogenetic analysis was conducted based on the complete plastid genomes of this species and other 13 species belonging to Iridaceae, Amaryllidaceae, Asparagaceaeand and Asphodelaceae. The sequences were aligned with MAFFT (Katoh and Standley 2013). The maximum-likelihood (ML) and Bayesian inference (BI) phylogenetic trees were reconstructed using RAxML (Stamatakis 2014) and MrBayes (Ronquist et al. 2012). The phylogenetic tree (Figure 1) indicated that four species from *Iris* (included *I. domestica*) formed a monophyletic group, and *I. domestica* was closer to *I. gatesii*. This reported complete plastid genome of this important Chinese medicine will provide useful information for the phylogenetic and evolutionary studies in *Iris* and Iridaceae.

**Figure 1.**
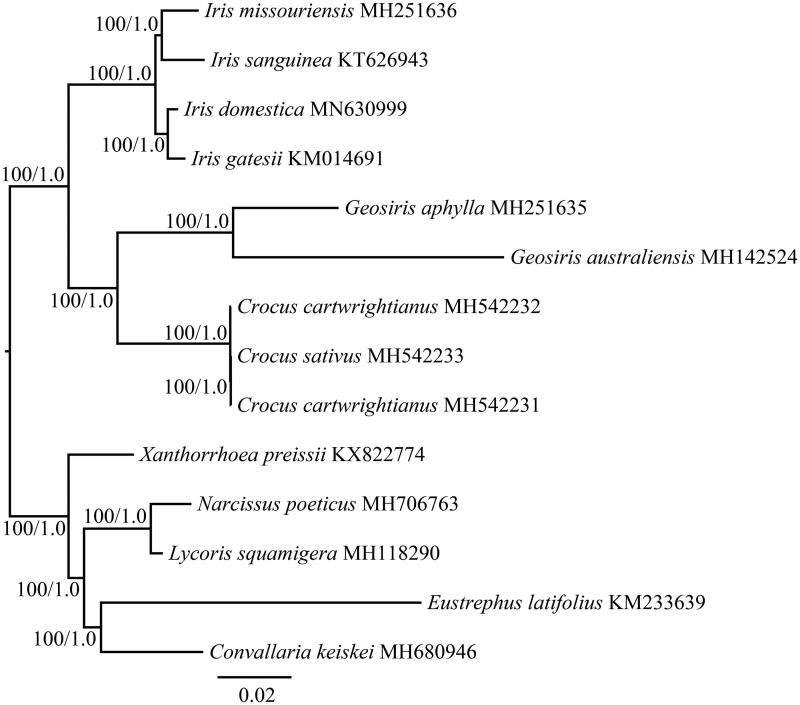
The maximum likelihood (ML) tree of Iridaceae inferred from the complete plastid genome sequences. Numbers at nodes correspond to ML bootstrap percentages (1,000 replicates) and Bayesian inference (BI) posterior probabilities.
